# Graphene Sheets with Defined Dual Functionalities for the Strong SARS‐CoV‐2 Interactions

**DOI:** 10.1002/smll.202007091

**Published:** 2021-02-02

**Authors:** Ievgen S. Donskyi, Chuanxiong Nie, Kai Ludwig, Jakob Trimpert, Rameez Ahmed, Elisa Quaas, Katharina Achazi, Jörg Radnik, Mohsen Adeli, Rainer Haag, Klaus Osterrieder

**Affiliations:** ^1^ Institut für Chemie und Biochemie Freie Universität Berlin Takustr. 3 14195 Berlin Germany; ^2^ BAM – Federal Institute for Material Science and Testing Division of Surface Analysis, and Interfacial Chemistry Unter den Eichen 44‐46 12205 Berlin Germany; ^3^ Institut für Virologie Robert von Ostertag‐Haus Zentrum für Infektionsmedizin Freie Universität Berlin Robert‐von‐Ostertag‐Str. 7‐13 14163 Berlin Germany; ^4^ Forschungszentrum für Elektronenmikroskopie and Core Facility BioSupraMol Institut für Chemie und Biochemie Freie Universität Berlin Fabeckstr. 36a 14195 Berlin Germany; ^5^ Department of Chemistry Faculty of Science Lorestan University Khorramabad Iran; ^6^ Department of Infectious Diseases and Public Health Jockey Club College of Veterinary Medicine and Life Sciences City University of Hong Kong Kowloon Tong Hong Kong

**Keywords:** graphene, graphene‐based polyglycerol sulfates, SARS‐CoV‐2 inhibitor, virucidality

## Abstract

Search of new strategies for the inhibition of respiratory viruses is one of the urgent health challenges worldwide, as most of the current therapeutic agents and treatments are inefficient. Severe acute respiratory syndrome coronavirus 2 (SARS‐CoV‐2) has caused a pandemic and has taken lives of approximately two million people to date. Even though various vaccines are currently under development, virus, and especially its spike glycoprotein can mutate, which highlights a need for a broad‐spectrum inhibitor. In this work, inhibition of SARS‐CoV‐2 by graphene platforms with precise dual sulfate/alkyl functionalities is investigated. A series of graphene derivatives with different lengths of aliphatic chains is synthesized and is investigated for their ability to inhibit SARS‐CoV‐2 and feline coronavirus. Graphene derivatives with long alkyl chains (>C9) inhibit coronavirus replication by virtue of disrupting viral envelope. The ability of these graphene platforms to rupture viruses is visualized by atomic force microscopy and cryogenic electron microscopy. A large concentration window (10 to 100‐fold) where graphene platforms display strongly antiviral activity against native SARS‐CoV‐2 without significant toxicity against human cells is found. In this concentration range, the synthesized graphene platforms inhibit the infection of enveloped viruses efficiently, opening new therapeutic and metaphylactic avenues against SARS‐CoV‐2.

## Graphical abstract

Present work shows that both electrostatic and hydrophobic interactions play a key role at the graphene/coronavirus interfaces and such materials can efficiently protect cells from coronavirus infections. While electrostatic interactions are responsible for the capturing of viruses, hydrophobic long alkyl chains can disrupt the envelop of the captured virions via hydrophobic interactions leading to a dual effect.

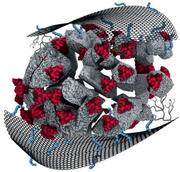

SARS‐CoV‐2 poses a major threat to the public health worldwide, as it causes a respiratory disease named COVID‐19. Since the first case report in December 2019, a pandemic ensued with approximately one million deaths within the first nine months.^[^ ^1^ ^]^ SARS‐CoV‐2 belongs to the beta‐coronavirus genus. All coronaviruses have a lipid envelope with a capsid, which encapsulates the helical nucleocapsid with the RNA genome.^[^ ^2^ ^]^ The most prominent viral envelope component is the spike glycoprotein (S), which interacts with the angiotensin‐converting enzyme 2 (ACE2) on the surface of host cells and initiates virus entry, the first step of the SARS‐CoV‐2 infection cycle.^[^ ^1^, ^3^, ^4^, ^5^ ^]^ Moreover, the polybasic cleavage site of S is found to play a crucial role in the binding between the virus and ACE2.^[^ ^6^ ^]^ Numerous efforts have been devoted to development of vaccines that generate neutralizing antibodies toward S to block viral interaction with ACE2.^[^ ^7^, ^8^, ^9^ ^]^ Similarly, the isolation and production of neutralizing antibodies targeting S promote the therapeutic treatment of SARS‐CoV‐2 infections, underscoring the importance of S as a target to block infection.^[^ ^10^, ^11^, ^12^ ^]^ However, it is also noticed that SARS‐CoV‐2 can mutate and it is conceivable that only small mutation of S by could render previously neutralizing antibodies ineffective.^[^ ^13^, ^14^, ^15^ ^]^ Therefore, it is essential to consider a new target instead of the S to develop materials for a broad‐spectrum inhibition of coronaviruses.

Irreversible rupturing of the lipid viral envelope is a potential new approach for SARS‐CoV‐2 inhibition. Most of the surface‐active reagents, e.g., sodium dodecyl sulfonate, exhibit strong interactions with cellular membranes and can also rupture viral envelopes leading to inactivation of the virus, a so‐called virucidal compound. However, since they also act against the cellular membranes, they are highly toxic and can only be used for ex vivo disinfection of surfaces and hands.^[^ ^16^, ^17^ ^]^ Hence, it is necessary to find a less toxic alternative for SARS‐CoV‐2 inhibition. Earlier reports have pointed out that negatively charged amphiphilic structures show broad‐spectrum inhibitory activity via the interaction with the viral envelope, including but not limited to herpes simplex virus (HSV), respiratory syncytial virus, human metapneumovirus, hepatitis C virus, ZIKA virus, etc.^[^ ^18^, ^19^ ^]^ Surprisingly, in these studies with a fine tuning of charges and hydrophobicity, these compounds show low cytotoxicity but high virus inhibitory activity. The reason for such an observation could be due to the fast curing of membrane damages by self‐repairing mechanisms.^[^ ^20^ ^]^ Therefore, it is believed that the concept of envelope rupturing can be used to develop a robust SARS‐CoV‐2 inhibitor.^[^ ^21^ ^]^

Graphene, as a flexible 2D nanomaterial, is a promising candidate for the development of virus inhibitors due to the facilitated multivalent interactions at the functionalized interface.^[^ ^22^, ^23^ ^]^ Modified graphene nanoplatforms have shown significant inhibition of various pathogens due to their high binding affinity for bacteria and viruses.^[^ ^24^, ^25^, ^26^, ^27^, ^28^, ^29^, ^30^ ^]^ Even though the possible mechanisms of the interactions between graphene derivatives and pathogens are still a controversial topic,^[^ ^25^, ^31^ ^]^ there are several suggested pathways for the antipathogenic action of these nanomaterials. Graphene materials are able to capture the pathogens by interactions with specific antibodies or ligands,^[^ ^32^, ^33^ ^]^ electrostatic interactions,^[^ ^34^, ^35^, ^36^ ^]^ trapping^[^ ^37^, ^38^ ^]^ or wrapping.^[^ ^39^, ^40^ ^]^ Furthermore, graphene sheets can destroy the pathogens by hydrophobic^[^ ^23^, ^41^, ^42^ ^]^ or mechanical interactions.^[^ ^43^, ^44^ ^]^ The versatility of nanoplatforms has been shown by their inhibition of various viruses, exemplified by designed inhibitors for HSV, African swine fever virus, alpha HSV, pseudorabies virus, and other viruses.^[^ ^23^, ^35^, ^39^, ^45^ ^]^ In previous studies, graphene‐based compounds have been active against viruses mostly via a “binding and wrapping mechanism,” and have been virustatic inhibitors. Consequently, the virus is only temporarily inhibited from the interaction with host cells and the virus can escape from the inhibitor.^[^ ^19^ ^]^

Herein, we report the development of graphene‐based virucidal compounds that rupture the envelopes of coronaviruses thus inhibiting viral infection irreversibly. The surface of graphene is functionalized with polyglycerol sulfate (PGS) and aliphatic chains of different length. In this study, we have investigated thoroughly how various sulfated graphene‐based materials interact with the virions of feline coronavirus (FCoV) and SARS‐CoV‐2 to explore the potential of designing virucidal graphene‐based inhibitors. A series of graphene platforms with PGS and alkyl amines of various chain lengths to surface of graphene is synthesized and compared for their inhibitory effect on coronaviruses. Functionalized graphene platforms that bear alkyl chains shorter than 10 carbon atoms show moderate infection inhibition without significant toxicity for cells. However, materials with longer aliphatic chains (>9 carbon atoms) indicate stronger inhibition and virion disruption of both coronaviruses, authentic SARS‐CoV‐2 and FCoV, but also exhibit higher toxicity for eukaryotic cells. Our results demonstrate a key role of electrostatics, i.e., polyglycerol sulfate coverage for virus capturing and a strong correlation between the length of alkyl chains and virucidal activity of such functionalized graphene‐based materials against coronaviruses.

In order to construct a platform for synergistic electrostatic and hydrophobic interactions with SARS‐CoV‐2 (**Figure** **1**a), graphene derivatives with polyglycerol sulfate coverage (G‐PGS) and dual alkyl amines/polyglycerol sulfate functionalities (G‐PGS‐C*x*; *x* is the number of carbon atoms in aliphatic chains) were produced by our recently reported methods (Figure 1b).^[^ ^23^, ^46^ ^]^ First, triazine was attached to the surface of graphene, which formed platforms with dichlorotriazine functional groups, and then polyglycerol with 5% amine functional groups was attached to the surface of these platforms. Next, the polyglycerol‐covered graphene sheets were converted into their polysulfated analog, forming graphene‐polyglycerol sulfate (G‐PGS) (Figure S1, Supporting Information). Each step of functionalization was proven using X‐ray photoelectron spectroscopy (XPS) due to the change in the elemental ratios both in survey and highly resolved C1s spectra (Figure S1b,c and Table S1, Supporting Information). Afterward, alkyl amines with various length of chains were attached to the triazine functional groups of G‐PGS and graphene materials with dual alkyl amine/polyglycerol sulfate functionalities (G‐PGS‐C*x*) were produced (Figure 1b)_._ The change in the ratio of C=C/C—O components in highly resolved C1s spectra during the functionalization steps (Table S1, Supporting Information) proved successful attachment of polyglycerol and alkyl amines to graphene surface. To explore the impact of the length of aliphatic chains on the antiviral activity of graphene platforms, alkyl amines with a different number of carbon atoms (*x* = 6, 9, 10, 11, and 12) were conjugated to the surface of G‐PGS. The charge of the various graphene derivatives was not affected due to the attachment of alkyl chains (**Table** **1**). According to the atomic force microscopy (AFM) measurements (Figure 1c–e), conjugation of long alkyl moieties to the surface of G‐PGS did not change lateral size of sheets, however G‐PGS‐C*x* height was significantly increased (10.5 nm for G‐PGS‐C12; 6.1 nm for G‐PGS‐C6) in comparison with G‐PGS (3.8 nm), according to the height and lateral size histograms of corresponding materials (Figure S2, Supporting Information).

**Figure 1:**
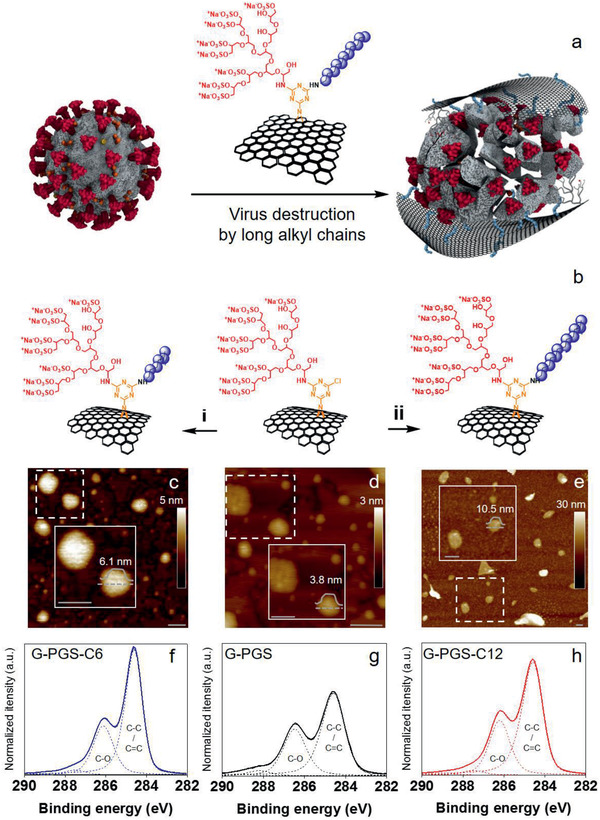
a) Schematic representation of intended interactions between G‐PGS‐C11 and SARS‐CoV‐2. While negatively charged polyglycerol sulfate interact with the positively charged domains of S on the surface of SARS‐CoV‐2, aliphatic chains penetrate into its membrane and disintegrate the virus. b) Schematic representation of the synthesis of G‐PGS‐C*x*. Synthesis of G‐PGS derivatives with i) short (C6, C9) and ii) long aliphatic chains (C10, C11, and C12). G‐PGS, DMF, C_6_H_13_NH_2_/C_9_H_19_NH_2_/C_10_H_21_NH_2_/C_11_H_23_NH_2_/C_12_H_25_NH_2_, triethylamine, 25–60 °C, 24 h. c–e) AFM images with enlarged parts showing typical size and height of graphene sheets, and highly resolved f–h) XPS spectra for G‐PGS‐C6, G‐PGS, and G‐PGS‐C12, respectively. Scale bars correspond to 100 nm.

**Table 1:** Zeta‐potential, cytotoxicity, and IC50 values of graphene platforms against both FCoV and SARS‐CoV‐2

Sample	Zeta‐potential mV	CC50_Vero E6_ [µg mL^−1^]	IC50_FCoV_ [µg mL^−1^]	IC50_SARS‐CoV‐2 _[µg mL^−1^]
G‐PGS	−46 ± 5	>1000	>1000	>1000
G‐PGS‐C6	−46 ± 5	>1000	>1000	>1000
G‐PGS‐C9	−49 ± 7	>1000	749.4 ± 107.8	339.7 ± 128.8
G‐PGS‐C10	−56 ± 11	63.4 ± 35.8	9.8 ± 10.2	29.1 ± 12.2
G‐PGS‐C11	−54 ± 8	68.9 ± 26.4	6.3 ± 1.2	0.8 ± 0.3
G‐PGS‐C12	−56 ± 10	100.1 ± 40.2	85.2 ± 50.9	22.9 ± 7.4

The cytotoxicity of the functionalized graphene sheets against lung epithelial cells (A549), lung bronchial epithelial cells (HBE) and kidney Vero E6 cells was investigated by assessing the cells viability with the colorimetric assay Cell Counting Kit‐8 (CCK‐8). While samples with long aliphatic chains (≥10) reduced the cell viability at 50 µg mL^−1^ considerably, the samples with shorter aliphatic chains (<10) did not show significant toxicity against the investigated cells lines (Table 1). It is presumed to be caused by the different interactions between the functionalized graphene platforms with different alkyl chains and the cell membranes. The functionalized graphene platforms with long aliphatic chains (≥10) could penetrate the cellular membrane to induce cell death, while their counterparts with shorter alkyl chains were not able to diffuse into the membrane of the host cell. Therefore, it is reasonable to presume that long aliphatic chains are needed for efficient inactivation of viral particles (Table 1 and Figures S3 and S4: Supporting Information). These outcomes are in line with our earlier findings on graphene/pathogen interfaces, where electrostatic interactions were capturing factor and hydrophobic interactions were rapturing factor.^[^ ^23^, ^47^, ^48^ ^]^

Next, we studied inhibitory effects of the functionalized graphene platforms with FCoV, a coronavirus strain infecting cats. Though the S of FCoV and SARS‐CoV‐2 are slightly different, their envelope structures are similar, qualifying FCoV to be a surrogate to SARS‐CoV‐2 for inhibition studies. Plaque reduction assay showed dose‐dependent inhibition efficiency for the incubated compounds (**Figure** **2**a). In line with cellular interaction studies, the functionalized graphene platforms with longer aliphatic chains showed stronger virus inhibition than their counterparts with shorter alkyl chains. The transition domain for effective virus inhibition was the chain length from C9 to C10, the half maximal inhibitory concentration (IC50) values of G‐PGS‐C9 (749.4 ± 107.8 µg mL^−1^) and G‐PGS‐C10 (9.8 ± 10.2 µg mL^−1^) (Table 1 and Figure 2b). G‐PGS‐C11 was the most effective inhibitor and showed potent inhibition toward FCoV with an IC50 value of 6.3 ± 1.2 µg mL^−1^, highlighting the necessity of long aliphatic chains for virus inhibition. IC50 values of G‐PGS‐C12 toward FCoV were lower than for G‐PGS‐C11 (85.2 ± 50.9 µg mL^−1^). Therefore, material with the aliphatic chain length consisting of 11 carbon atoms showed the optimum values for the virus inhibition.

**Figure 2:**
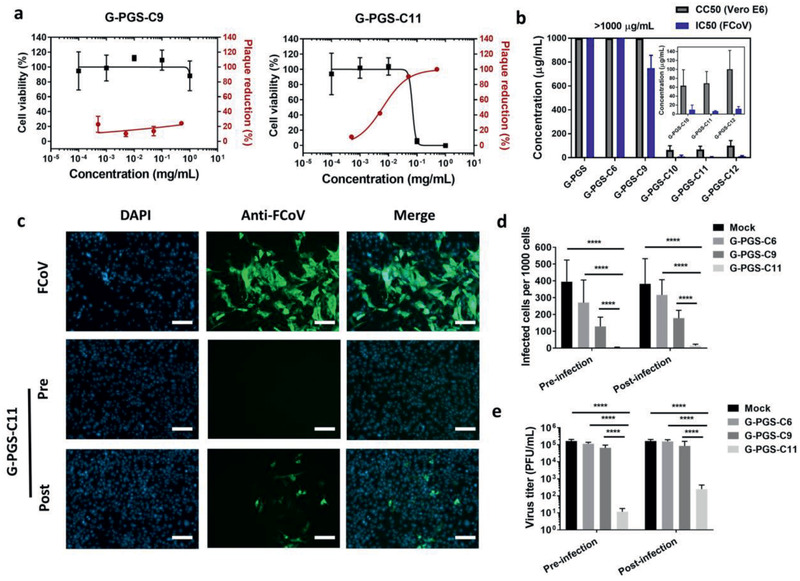
a) Cell viability versus plaque reduction (FCoV) curves of the functionalized graphene platforms at different concentrations. b) Comparison between CC50 (Vero E6 cells) with the IC50 (FCoV) values for the compounds. c) Immunofluorescent images for the FCoV‐infected cells in the presence of G‐PGS, G‐PGS‐C9 and G‐PGS‐C11 at 10 µg mL^−1^. Scale bar: 100 µm. The images for other compounds are shown in the Supporting Information. d) Counting of infected cells by FCoV in the presence of G‐PGS, G‐PGS‐C9, and G‐PGS‐C11 at 100_ _µg mL^−1^. e) Titration of virions in the medium from the pre‐ and post‐infection studies. Values are expressed as mean ± SD, *n* = 4. ****p < 0.001 from student's *t*‐test.

We further investigated the ability of the compounds to protect the cells from the infection by FCoV. The cells were first incubated with the compounds for 1 h and then infected with FCoV at a multiplicity of infection (MOI) of 0.1, a procedure we refer to as pre‐infection treatment. After 24 h of infection, the cells were stained by anti‐FCoV antibodies to determine infection (Figure 2c–e). The number of infectious virus in the medium was studied by plaque reduction assays and expressed as plaque‐forming units per mL (PFU mL^−1^) to quantitate virus replication (Figure 2e). Without treatment, robust infection was observed by FCoV and the virus titer reached ≈2 × 10^5^ PFU mL^−1^. Pre‐treatment of cells with the compounds reduced FCoV infection. G‐PGS‐C11 nearly abolished the infection in the pretreatment test. There were no infected cells observed, at our detection limit, and only 10 PFU mL^−1^ of FCoV were detected in the medium, corresponding to an inhibitory effect higher than 99.99%. These results indicated that G‐PGS‐C11 could be a potent prophylactic agent to prevent coronavirus infections.

In order to study the inhibition of virus replication by functionalized graphene sheets after internalization into the cells, cells were first infected and then incubated with the graphene derivatives, which we refer to as post‐infection treatment. Infection efficiency and virus replication were studied by antibody staining and plaque assay. In this case G‐PGS‐C6 and G‐PGS‐C9 showed poor inhibition. Even though the number of infected cells decreased slightly, the virus titer in the medium was at the comparable level as the control experiment. However, G‐PGS‐C11 showed clear inhibition in the post‐infection treatment study. Only a few infected cells, which were probably infected before treatment with compounds, were observed. Virus replication was reduced to ≈100 PFU mL^−1^, corresponding to an inhibitory effect higher than 99.9%. The cells also survived from the treatment, as the number of cells was not decreased, indicated by Hoechst 32 253 staining (Figure 2e and Figure S5: Supporting Information). The activity of G‐PGS‐C11 in the post‐infection treatment study indicated that it could be a therapeutic agent toward coronavirus infection.

The inhibition of virus by the functionalized graphene platforms was further investigated by a virucidal assay. It should be mentioned that most inhibitors act through a virustatic mechanism. They inhibit virus infection through competitive binding of the virus to its receptor.^[^ ^12^, ^49^, ^50^ ^]^ In this case, virions are only blocked from entry into host cells instead of being completely deactivated. Upon dilution of the inhibitor below its IC50, the virions may be released from the inhibitors and are able to start the infection again.^[^ ^18^, ^19^ ^]^ To ensure the final deactivation of virions, virucidal compounds that damage virions irreversibly should be developed, especially for the highly infectious SARS‐CoV‐2. To test the virucidal potential of the compounds, the FCoV virions (>10^6^ PFU) were first incubated with the compounds a 1000 times higher concentration than their IC50 values for 1 h. After dilution to a concentration 10‐times lower than their IC50, the number of active virions was determined by plaque assay (**Figure** **3**a). Samples, which showed a clear reduction of active virions even below the IC50 values, were considered as virucidal compounds.

**Figure 3:**
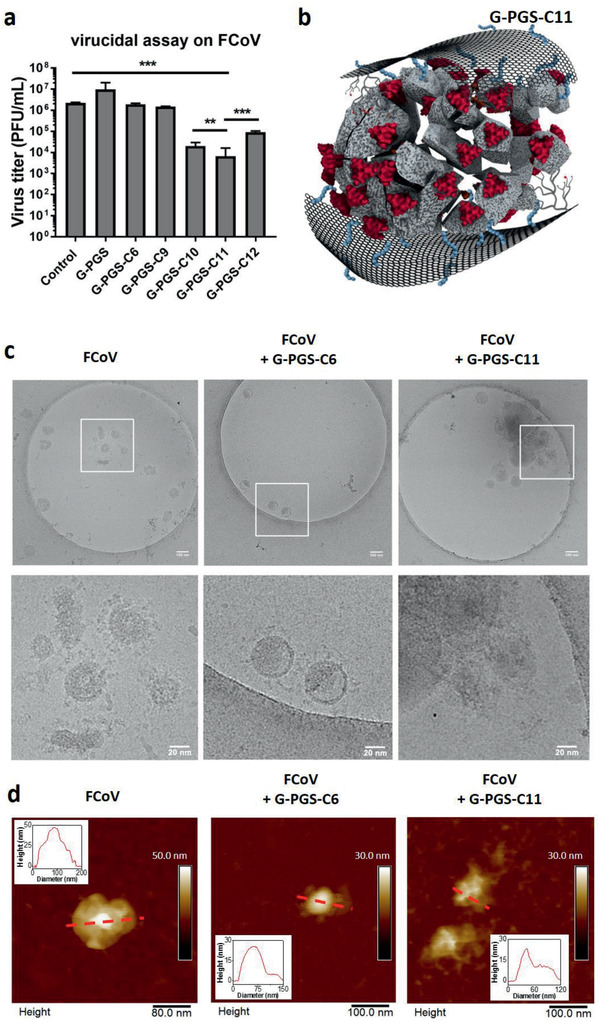
a) Virucidal assay for the functionalized graphene platforms. Values are expressed as mean ± SD, *n* = 4. b) Schematic illustration of virus rupturing by G‐PGS‐C11. c) Cryo‐EM images for pristine FCoV virions and FCoV virions incubated with G‐PGS‐C6 and G‐PGS‐C11 for 1 h. d) AFM images of FCoV virion with clear S on its surface; FCoV virions incubated with G‐PGS‐C6 and G‐PGS‐C11 for 1 h.

Similar to the results of the plaque reduction assays, a clear transition from virustatic to virucidal was observed at C9‐C10_._ While samples with aliphatic chains <10 (G‐PGS, G‐PGS‐C6, and G‐PGS‐C9) were virustatic, the platforms with aliphatic chains ≥ 10 (G‐PGS‐C10, G‐PGS‐C11, and G‐PGS‐C12) were virucidal. G‐PGS‐C11 was considered as the best material with two orders of magnitudes of virus titer reduction. The results of the plaque reduction assay and the virucidal assays showed that aliphatic chains more than 9 carbon atoms on the surface of graphene were necessary for virus membrane rupture (Figure 3b). G‐PGS‐C11 showed the best activities for both virus inhibition and virucidality, indicating that in our system, aliphatic chain with 11 carbon atoms was the optimal.

To reveal the virucidality of the compounds and investigate the influence of functionalized graphene platforms on viruses under native (hydrated) conditions, we used cryogenic transmission electron microscopy (cryo‐EM). Purified FCoV virions were incubated with G‐PGS‐C6 (virustatic) and G‐PGS‐C11 (virucidal) for 1 h and fixed by 2.5% formaldehyde. The samples were then cryofixated by plunge freezing and microscopically analyzed in a 200 kV TEM under low‐dose conditions (Figure 3c). Pristine FCoV showed the typical morphology of coronavirus. The virions had a diameter of about 100 nm with visible S anchored on the surface of the envelope. We were not able to recognize the functionalized graphene platforms, probably because the ultra‐thin 2D material tends to generate a very low contrast. However, the virions incubated with G‐PGS‐C6 still exhibited the typical morphology of coronavirus with intact S on the surface of the envelope (Figure 3c). The observations suggested that the virustatic compound (G‐PGS‐C6) interacted with the virions without damaging them, at least no morphological changes were visible on the virions after incubation. In case of incubation with G‐PGS‐C11, the virions underwent morphological alterations (Figure 3c). Most noticeably, the virions disintegrated by rupturing the envelope and depleting the S. The observation correlates well with the results of virucidal assay where virions were inactivated upon interactions with G‐PGS‐C11.

To study the interactions of FCoV with graphene sheets further, the samples were analyzed by AFM in liquid conditions (Figure 3d and Figure S6: Supporting Information). AFM confirmed that the lateral dimensions of virions were in the same size range as in Cryo‐EM. S could be identified clearly in the topographic images of FCoV virions alone, as well as in case of FCoV virions that were incubated with G‐PGS‐C6. Moreover, G‐PGS‐C6 sheets interacting with the surface of FCoV virion could be observed (Figure 3d and Figure S6: Supporting Information). However, in case of G‐PGS‐C11 the virions were not intact anymore, and they lost their spherical morphology and were disrupted due to the interactions with graphene sheets that covered the virions (Figure 3d and Figure S6: Supporting Information). Taken together, the results confirmed our hypothesis that the negatively charged PGS bound electrostatically to the positive patches on the S,^[^ ^51^ ^]^ and aliphatic chains interacted with the viral envelope (Figures 1a and 3b). The lipid interaction enabled the rupture of the viral envelope and consequently the inactivation of the virus. The 2D flexible structure of graphene further facilitated multivalent interactions with the virus and enhanced both virus binding and incapacitation.^[^ ^43^ ^]^

The half maximal cytotoxic concentration (CC50) and FCoV IC50 for G‐PGS‐C11 were 42.6 ± 30.1 and 4.7 ± 0.7 µg mL^−1^, respectively. The range between these two concentrations represents a therapeutic window that could be considered for virus inhibition and incapacitation without serious damaging of host cells. As shown on Figure 2b, in both pre‐ and post‐infection treatment studies, the treatment by G‐PGS‐C11 at 10 µg mL^−1^ eliminates FCoV infection without showing cellular toxicity. The difference between virus inhibition and cellular toxicity may be explained by the surface curvature of biosystems. A virus is only 100 nm in diameter, while a cell normally exhibits a surface of 10 µm. Therefore, the membrane curvature and strain are higher in the virus compared to the cells. The self‐repairing machinery also enables the cells to tolerate membrane damage and to restore the membrane.^[^ ^20^ ^]^ The virus particles mainly contain genetic information but no enzymes or other structures, thereby lacking the ability to repair membrane damage by virucidal inhibitors.

Finally, the inhibitory effect of the functionalized graphene platforms on authentic SARS‐CoV‐2 was studied using a plaque reduction assay (**Figure** **4**a,b). Similar to the results of FCoV, the compounds with long aliphatic chains showed the strongest inhibition. Alkyl chains with more than 9 carbon atoms were necessary for the inhibition of SARS‐CoV‐2. G‐PGS‐C11 showed the strongest inhibition with an IC50 of 0.8 ± 0.3 µg mL^−1^, with a selectivity index (ratio of CC50 to IC50) of 86. Although G‐PGS‐C11 showed toxicity at high concentrations, the potent inhibition of SARS‐CoV‐2 at doses much lower than its CC50 value may allow safe antiviral treatment with this inhibitor. For example, G‐PGS‐C11 showed clear virus inhibition at 50 µg mL^−1^, a concentration where this compound did not show significant cellular toxicity. As shown in the image of the plaque reduction assays of Figure 4a, at this concentration the base cell layer remained intact, indicating no cellular toxicity. For G‐PGS‐C11, the virions remained inhibited upon dilution, indicating that the virions were inhibited irreversibly. Therefore, G‐PGS‐C11 was considered virucidal to SARS‐CoV‐2, due to interactions with viral envelope. The other functionalized graphene platforms (G‐PGS, G‐PGS‐C6, and G‐PGS‐C9) were virustatic instead of virucidal (Figure 4c).

**Figure 4:**
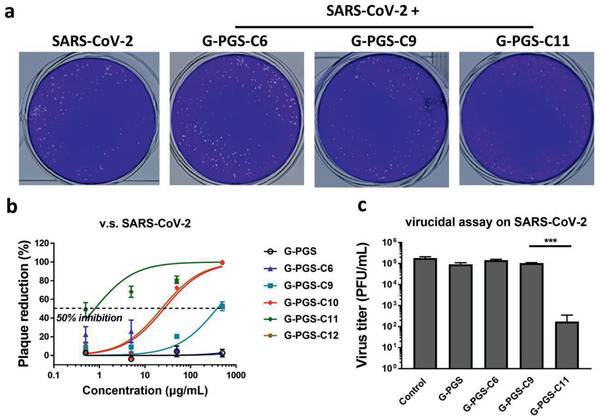
a) Typical plaque images for SARS‐CoV‐2 treated by the functionalized graphene platforms at 50 µg mL^−1^. b) Plaque reduction curves for SARS‐CoV‐2 in the presence of the functionalized graphene platforms at different concentrations. c) Virucidal assay for the functionalized graphene platforms. Values are expressed as mean ± SD, *n* = 4.

We successfully synthesized graphene platforms with defined dual polyglycerol sulfate/aliphatic chains functionalities for SARS‐CoV‐2 binding and disintegration. In contrast with previously reported virustatic graphene‐based inhibitors, the synthesized inhibitors in this study were found to be virucidal. Virions bound to the functionalized graphene sheets by multivalent electrostatic interactions with the negatively charged PGS were disintegrated by the alkyl chains. Disruption of coronavirus envelopes occurred with elongation of aliphatic chains from G‐PGS‐C9 to G‐PGS‐C10 and showed the strongest inhibition and virucidality against feline coronavirus with G‐PGS‐C11. Therefore, alkyl chains should be long enough to exert efficient disintegration of the viral envelope. To rupture the envelop of the virus, the negatively charged PGS branches first interacted with the positively charged patches of the virion, then the aliphatic chains ruptured the membrane of the viral envelop. Graphene acted as a 2D platform to facilitate the interactions with virus particles. G‐PGS‐C11 displayed the most efficient antiviral properties against authentic SARS‐CoV‐2 without showing significant toxicity against eukaryotic host cells. The observed post‐treated effect and selectivity index of 86 opens the door for potential therapeutic applications.

## Experimental Section

##### Plaque Reduction Assays

The compounds were 10‐fold diluted in Dulbecco's Modified Eagle's Medium (DMEM) and incubated with FCoV‐containing solutions with ≈100 PFU for 1 h at 37 °C. Afterward, the number of active virions was determined by plating the virus/compound mixture on a confluent monolayer of CRFK cells. Virus adsorption was allowed for 1 h before the supernatant was aspirated and replaced by a semi‐solid overlay of 1.3% methylcellulose (viscosity 400 cP). The overlay was removed after 48 h; cells were fixed with 4% formalin and stained with FCoV‐specific antibodies (primary antibody: mouse anti‐FCoV, Bio‐Rad, cat# MCA2194; secondary antibody: Goat anti‐Mouse IgG Alexa Fluor 488, Invitrogen, cat# A32723) to visualize plaques. The plaque reduction ratio was determined as following

(1) $$\begin{array}{c} \text{Plaque}\;\;\text{reduction}\;\left(\%\right)\;\;=\;\;\left(1\;-\;\frac{\text{Plaque}\;\text{number}\;\left(\text{sample}\right)}{\text{Plaque}\;\text{number}\;\left(\text{virus}\;\text{control}\right)}\right)\;\times \;100\% \end{array}$$

##### Virucidal Assay

The functionalized graphene platforms (1 mg mL^−1^) were incubated with FCoV suspension containing ≈1 × 10^5^ PFU for 1 h at 37 °C. Afterward, the mixture was diluted in DMEM medium 10‐foldly 5 times to an endpoint of no active virions. The number of active virions was determined by plaque assay and virus titers were calculated back by the respective dilutions.

##### Plaque Reduction Assay and Virucidal Assay on SARS‐CoV‐2

SARS‐CoV‐2 München (SARS‐CoV2M; BetaCoV/Germany/BavPat1/2020) was propagated in Vero E6 cells and titrated via plaque assay.^[^ ^52^ ^]^ The plaque reduction assay and virucidal assay were performed in the same procedure as for FCoV, except that the plaques were stained by 0.75% crystal violet. The experiment was performed within the BSL3 lab in the Institute of Virology, Department of Veterinary Medicine at the Freie Universität Berlin.

## Conflict of Interest

The authors declare no conflict of interest.

## Supporting information

Supporting Information

## Data Availability

Research data are not shared.
