# Supplementary material for: Graphene Sheets with Defined Dual Functionalities for the Strong SARS‐CoV‐2 Interactions
Source: Small. 2021 Feb 2;17(11):2007091. doi: 10.1002/smll.202007091 (PMC7995151; doi:10.1002/smll.202007091)
Supplement: Supplementary file 1 — Supporting Information [file SMLL-17-2007091-s001.pdf]

© 2021 Wiley-VCH GmbH

NANO MICRO
small

Supporting Information

for *Small*, DOI: 10.1002/smll.202007091

Graphene Sheets with Defined Dual Functionalities for the Strong SARS-CoV-2 Interactions

Ievgen S. Donskyi, Chuanxiong Nie,* Kai Ludwig,* Jakob Trimpert,* Rameez Ahmed, Elisa Quaas, Katharina Achazi, Jörg Radnik, Mohsen Adeli, Rainer Haag, and Klaus Osterrieder*

WILEY-VCH

© 2021 Wiley-VCH GmbH

Supporting Information

Graphene Sheets with Defined Dual Functionalities for the Strong SARS-CoV-2 Interactions

Ievgen S. Donskyi,[‡,] Chuanxiong Nie,[‡,*] Kai Ludwig,[*] Jakob Trimpert,[*] Rameez Ahmed, Elisa Quaas, Katharina Achazi, Jörg Radnik, Mohsen Adeli, Rainer Haag, Klaus Osterrieder*

[‡] These authors contributed equally.

Materials

Graphite powder (median diameter 7-10 microns) was purchased from ACROS Organics. Glycidol (Sigma-Aldrich) was dried over CaH_2, distilled before use, and stored at 4 °C. Sodium hypochlorite (11-14 % available chlorine), methanesulfonyl chloride (MsCl) (≥ 99.7 %), triphenylphoshine (PPh_3) (99 %), sulfur trioxide pyridine complex ($Py^{•}SO_3^{-}$) (98 %), poly-L-Lysine solution (MW: 75,000-120,000) phosphate buffer solution 1.0 M, pH 7.4 (25 °C), hydrogen peroxide (30 % solution) (H_2O_2), triethylamine (99.5 %) (TEA), N,N-dimethylformamide (DMF) (≥ 99.8 %), 1-methyl-2-pyrrolidinone (NMP) (99.5 %), 1-hexylamine (C_6H_{13}-NH_2) (99 %), 1-nonylamine (C_9H_{19}-NH_2) (99.5 %), 1-decylamine ($C_{10}H_{21}$-NH_2) (≥ 99.0 %), 1-undecylamine ($C_{11}H_{23}$-NH_2) (98 %), 1-dodecylamine ($C_{12}H_{25}$-NH_2) (97 %) were purchased from Sigma Aldrich. 2,4,6-trichloro-1,3,5-triazine (TCT) (99 %) and sodium azide (NaN_3) (99 %) were purchased from Acros Organics. Sodium hydroxide (NaOH) (99 %) was purchased from Fisher Chemical. Anhydrous solvents were either obtained from solvent purification (MBraun MB-SPS-800) system or purchased as ultra-dry solvents from Acros Organics. Water was derived from a Milli-Q advantage A10 water purification system in all experiments. Biotech cellulose ester dialysis bags MWCO 20 kDa were purchased from Spectrum labs. All chemical compounds were used without further purification.

Methods

Zeta-potential measurements. Zeta-potential experiments were performed on a Malvern Zetasizer nano machine at 25 °C. Millipore water was used in all experiments. Measurements were performed with a Malvern-folded capillary zeta cell in automatic mode.

X-ray photoelectron spectroscopy (XPS) experiments. Gold substrates used for analysis of samples deposited thereon were cleaned in piranha solution (1:4) 30 % H_2O_2: 98 % H_2SO_4 (v/v) during ultrasonication at room temperature for 10 min. Then they were washed with the DI water 5 times and with acetone 2 times. After drying overnight, the studied compounds were dissolved in methanol and evenly distributed dropwise across the surface of gold substrates. XPS spectra were recorded using a Kratos Axis Ultra DLD spectrometer equipped with a monochromatized Al Kα X-ray source (1486.69 eV) using an analyzer pass energy of 80 eV for survey spectra that were used for quantification. High-resolution, core-level O1s, C1s, and N1s spectra were recorded in FAT (fixed analyzer transmission) mode at a pass energy of 20 eV. Both the electron emission angle and the source-to-analyzer angle were 60 °. The binding energy scale of the instrument was calibrated following a Kratos Analytical Ltd procedure that used ISO 15472 binding energy data. Spectra were recorded by setting the instrument to the hybrid lens mode and the slot mode, which provided approximately a 300 x 700 μm^2 analysis area and using charge neutralization. All XPS spectra were processed with the UNIFIT program (version 2017). A Gaussian/Lorentzian product function peak shape model GL (30) was used in combination with a Shirley background. If not otherwise denoted, the L-G mixing for component peaks in all spectra were constrained to the value of 0.39. Peak fitting of C1s spectra was performed by using an asymmetric peak shape model for the graphene C1s' component peak and a symmetric peak shape model for all other component peaks. After peak fitting of the C1s spectra, all the binding energies were calibrated in reference to the graphene C1s component at a binding energy of 284.6 eV.

Atomic Force Microscopy (AFM). Samples were prepared by spin-casting dispersions of graphene materials (0.1 mg/mL) in deionized water onto freshly cleaved mica substrates at 50 rps for 60 seconds. The mica substrates were mounted onto Ø12 mm stainless steel discs with double-sided tape. Measurements were performed using a Bruker Multimode 8, Nanoscope 5 with a J-type scanner, operated in tapping mode with Nanosensors PPP-NCLR cantilever tips with a typical resonant frequency of 190 kHz, a spring constant of 48 N m^{-1}, and a tip radius of < 10 nm. Images were recorded at a minimum resolution of 1024 x 1024 and a scan speed of 0.8 Hz or lower. All experiments were conducted under ambient conditions, and results were analyzed using the Bruker NanoScope Analysis 1.8 software, along with Gwyddion. Images were line-flattened using a first order (linear) fit.

Cell viability assay. Vero cells were incubated with different functionalized graphene platforms for 24 h, and cell viability was determined using the WST-1 assay (Cayman Chemicals).[44] Briefly, cells were grown in 96-well plates and incubated with functionalized graphene platforms for 1 h at 4 °C. The plates were moved to the incubator at 37 °C and further incubated for 24 h. The WST-1 mixture was added to the cells and after 2 h incubation at 37 °C, absorbance of each sample was read using a microplate reader (Berthold Technologies) at 450 nm wavelength. Mock cells (untreated with graphene derivatives) and cells treated with 0.1 % hydrogen peroxide solution for 30 s were used as controls.

Propagation of FCoV. FCoV (ATCC VR-989, WSU 79-1683 (3)) is propagated on Crandell-Rees Feline Kidney (CRFK) cells (ATCC CCL-9). Briefly, the cells at 80 % confluency were infected by FCoV at MOI of 0.1 and cultured for 48 h. The virions in the supernatant were harvested by centrifugation at 400 rpm for 5 min. The virus containing supernatant was titrated by a plaque assay on CRFK cells and stored at –80 ^{o}C before use.

Cellular infection assay. CRFK cells were seeded in 24-well plates until they reached 90 % confluency. For the pre-infection treatment test, the cells were first treated with compounds at a dose of 10 μg/mL for 1 h at 37 ^{o}C and then infected by FCoV at a MOI of 0.1. For the post-

infection treatment test, the cells were first infected by FCoV at a MOI of 0.1 and then the compounds were added into the medium at the dose of 10 μg/mL. In both tests, the cells were cultured for another 24 h and the infected cells were stained with FCoV-specific antibodies (primary antibody: mouse anti-FCoV, Bio-Rad, cat# MCA2194; secondary antibody: Goat anti-Mouse IgG Alexa Fluor 488, Invitrogen, cat# A32723). The cell nucleus was stained by Hoechst 33342. The images were acquired on a Zeiss fluorescent microscope.

Cryo-EM imaging. FCoV were concentrated and purified by ultracentrifugation with 20 % sucrose solution. The purified virions were incubated with the functionalized graphene platforms for 1 h at 37 ^{o}C and fixed by 1 % glutaraldehyde for 1 h. 5 µL aliquots of the samples were applied to glow-discharged, perforated, carbon film-covered microscopical 200 mesh grids (R1/4 batch of Quantifoil, MicroTools GmbH, Jena, Germany) and vitrified by automatic blotting and plunge freezing with a FEI Vitrobot Mark IV (Thermo Fisher Scientific Inc., Waltham, Massachusetts, USA) using liquid ethane as cryogen. The vitrified specimens were transferred to a FEI TALOS ARCTICA electron microscope (Thermo Fisher Scientific Inc., Waltham, Massachusetts, USA). Micrographs were acquired on a FEI Falcon 3 direct electron detector.

AFM within a fluid chamber. Imaging of the interaction of graphene derivatives with FCoV to study binding was performed in PeakForce mode. A Multimode 8 Nanoscope from Bruker with a nanocontroller V was used in all experiments with an assembled fluid chamber. All measurements were carried out in PBS and at room temperature. Sample preparation is described as follows. Round discs of cleaved mica of about 1 cm in diameter were used as a substrate. The mica discs were glued with a double-sided tape onto circular metal pucks to be then mounted on the AFM head. 20 µL of 0.1 mg/mL poly-L-Lysine solution (Sigma-Aldrich MW: 75,000-120,000) was drop casted on the freshly cleaved mica surface to deposit thin film of polymer via electrostatic interactions. After 10 min of incubation the mica was rinsed with di-ionized water to remove unattached polymer. The mica was then allowed to dry. 5 µL

of FCoV solution or FCoV solution mixed with graphene derivatives were deposited at the center of the poly-L-Lysine-coated mica and allowed to rest for 5 min. Afterwards the mica surface was rinsed with PBS. Subsequently the sample was slightly blotted with filter paper without allowing it to dry, but only to reduce the amount of sample on the surface to a thin film. The sample was then immediately mounted on the AFM head and a closed fluid chamber was assembled. All measurements were performed in PBS. Sharp Nitride Lever tips with a nominal tip radius of 2 nm and spring constant of 0.35 N/m were used. Images were taken with a 256 point per line and 0.9 Hz scan rate.

Statistical analysis. All viability and plaque reduction assays were repeated three independent times. Statistical evaluation was performed using GraphPad Prism 5 (GraphPad software). One-way and two-way ANOVA was used to test for significance. Bonferroni adjustment was applied for multiple comparisons. Data represent mean values; standard deviations are indicated by error bars. P values less than 0.05 are considered significant.

Synthesis

*Synthesis of dichlorotriazine graphene (G-Trz). S*ynthesis of G-Trz was conducted according to our recently reported method (yield 78 %).[1]

Synthesis of polyglycerol with a few amino groups (PG-NH_2 5 %). First, polyglycerol was synthesized according to a reported procedure (yield 81 %).[2] M_n = 13.1 kDa. Polyglycerol with 5 % amino functional groups (PG-$NH_{2(5\ \%)}$) was synthesized according to the procedure reported in the literature.[3] The hyperbranched polyglycerol (PG, M_n = 13.1 kDa) was initially mesylated and subsequently azidated to convert the hydroxyl to azide functional groups with further reduction to the amino functional groups forming amino-functionalized PG (yield 60 %). *Polyglycerol-mesylate*. ^{1}H NMR (500 MHz, D_2O, δ): 4.2 – 3.4 (PG backbone), 3.3 – 3.2 (CH_3). *Polyglycerol-azide*. ^{1}H NMR (500 MHz, D_2O δ): 4.1 – 3.4 (PG backbone). *PG-amine with 5 % amino-functional groups*. ^{1}H NMR (500 MHz, D_2O δ): 4.0 – 3.3 (PG backbone).

Synthesis of graphene sheets with polyglycerol coverage (G-PG). Dispersion of G-Trz (0.2 g) in NMP (50 mL) was sonicated for 2 h and further stirred in an ice bath. Solution of PG-$NH_{2(5\ \%)}$ (0.96 g, 0.07 mmol) in NMP (10 mL) was added to G-Trz dispersion at 0 °C and stirred at same temperature for 3 h. TEA (21 µL, 0.15 mmol) was further added to the mixture and stirred for 1 h at 0 °C. The temperature of the mixture was increased to 25 °C and stirred for 2 days. Later, the mixture was dialyzed by a dialysis bag (cutoff MWCO 20 kDa) against water for 5 days and freeze-dried (yield 74 %).

Sulfation of G-PG (G-PGS). G-PG was sulfated according to the reported procedures in the literature.[10-12] Shortly G-PG (0.6 g) was dispersed in dry DMF (20 mL). Afterwards, pyridine sulfur trioxide (2.5 g, 15.7 mmol) in same solvent (20 mL) was added to the G-PG and stirred for other 24 h at 60 °C. Further, deionized water (20 mL) was added and the pH of the reaction mixture was adjusted to pH 9 using NaOH (20 % w/v), and the mixture was dialyzed using a dialysis bag (cutoff MWCO 20 kDa) against 1 M NaCl solution for 5 days, against deionized water for 7 days and in the end freeze-dried (yield 76 %).

Synthesis of G-PGS-Cx. Synthesis of G-PGS-Cx was performed according to our recently reported procedure (yield 78-82%).[4] Briefly, G-PGS (0.3 g) was dispersed in DMF (25 mL) and sonicated at room temperature for 30 minutes. Alkyl amines with different aliphatic chains (C_6H_{13}-NH_2, C_9H_{19}-NH_2, $C_{10}H_{21}$-NH_2, $C_{11}H_{23}$-NH_2, $C_{12}H_{25}$-NH_2) (42 µmol) were dissolved in DMF (15 mL) and added to the G-PGS dispersion at 25 °C. After stirring for 30 minutes at this temperature, triethylamine (5.6 µL, 0.042 mmol) was added to the reaction flask, mixture was heated till 60 °C, and stirred for 48 h. The product was dialyzed (MWCO 20 kDa) against water/isopropanol 1/1 mixture for 5 days. The solvent was evaporated, and the product was dried by lyophilization (yields 75-82%).

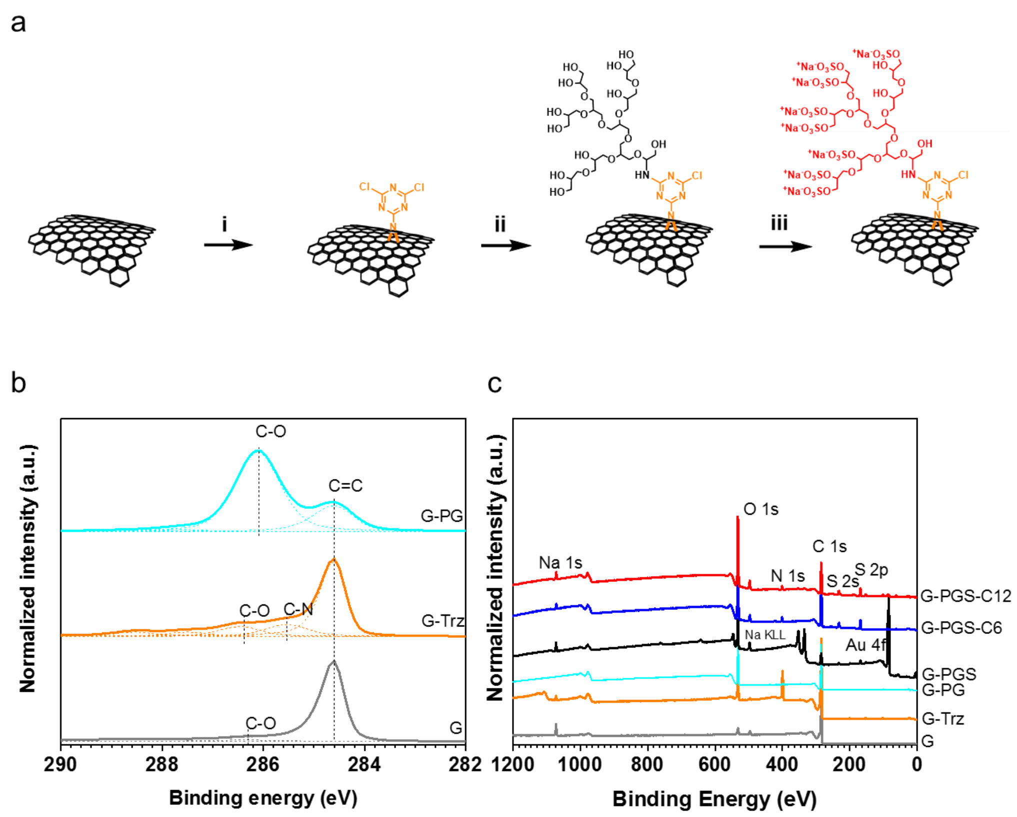

Figure S1. (a) Schematic representation of the synthesis of G-Trz, G-PG, and G-PGS. (i) NMP, TCT, sodium azide, 0 °C - 65 °C, 18 h. (ii) NMP, TEA, PG-NH_2, 0 °C - 25 °C, 48 h. (iii) DMF, Py*SO_3, 60 °C, 24 h. (b) Highly resolved XPS spectra for G, G-Trz, G-PG, respectively. (c) Survey spectra of G, G-Trz, G-PG, G-PGS, G-PGS-C6, G-PGS-C12. Highly resolved C1s XPS spectra of G-PGS, G-PGS-C6, G-PGS-C12 are shown in the main text, **Figure 1e-g**. For further XPS details see **Table S1**.

Table S1. Relative ratios of C=C/C–O components obtained by quantification of the XPS spectra displayed in **Figure S1a.**

Sample	C=C/C–O components ratio
G	48
G-PG	0.3
G-PGS	1.8
G-PGS-C6	2.6
G-PGS-C12	2.7

Relative ratios of C=C/C–O components was calculated dividing area of the component corresponding to C=C bond (binding energy of 284.6 eV) on the component corresponding to C–O bond (binding energy of ~ 286.2 eV) in the highly resolved C1s spectra of graphene materials.

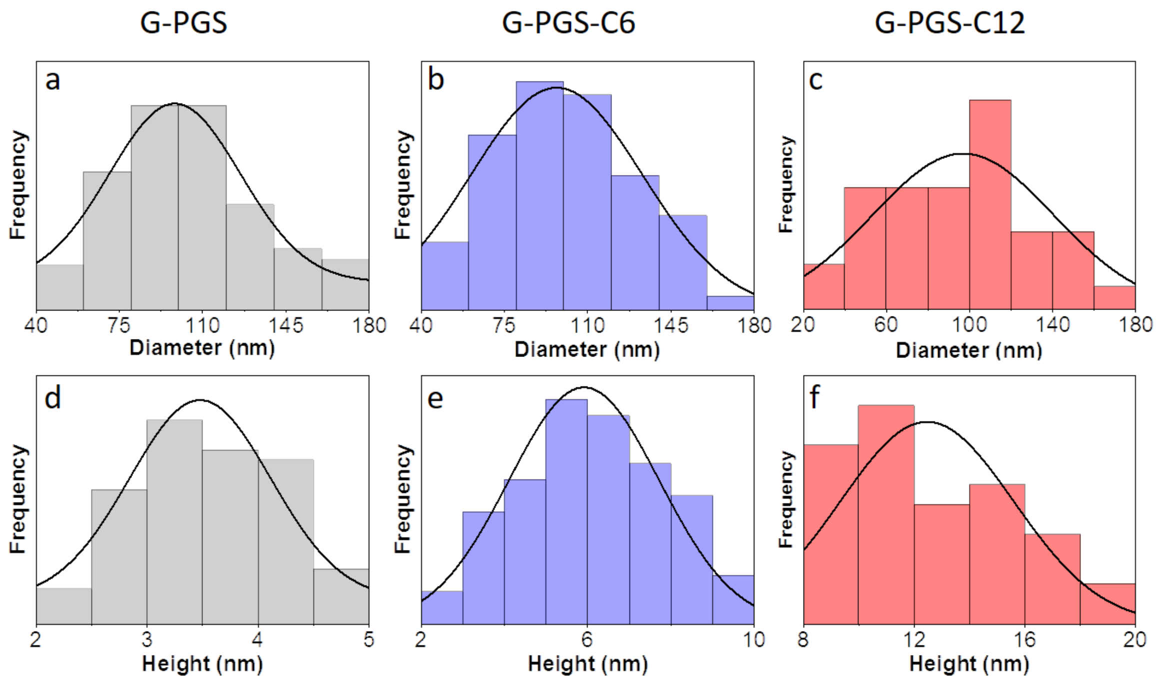

Figure S2. (a-c) Diameter and (d-f) height histograms for G-PGS, G-PGS-C6, and G-PGS-C12, respectively.

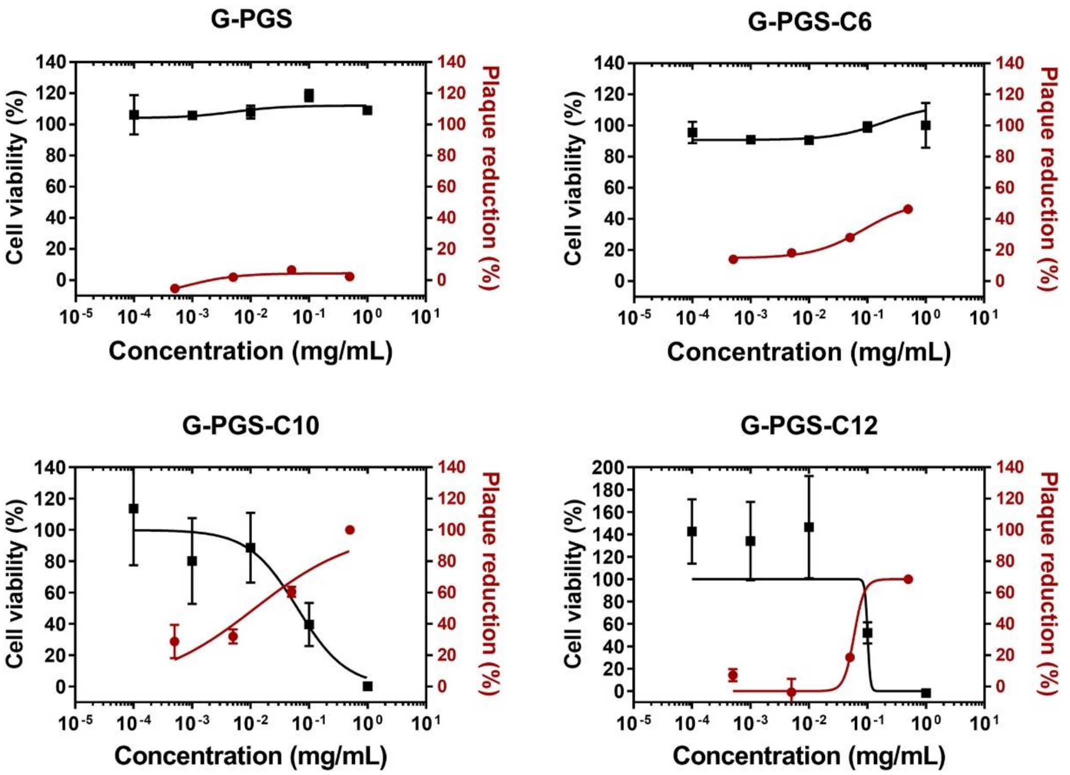

Figure S3. Cell viability for Vero cells vs. plaque reduction (FCoV) curves of the G-PGS, G-PGS-C6, G-PGS-C10, and G-PGS-C12 at different concentrations.

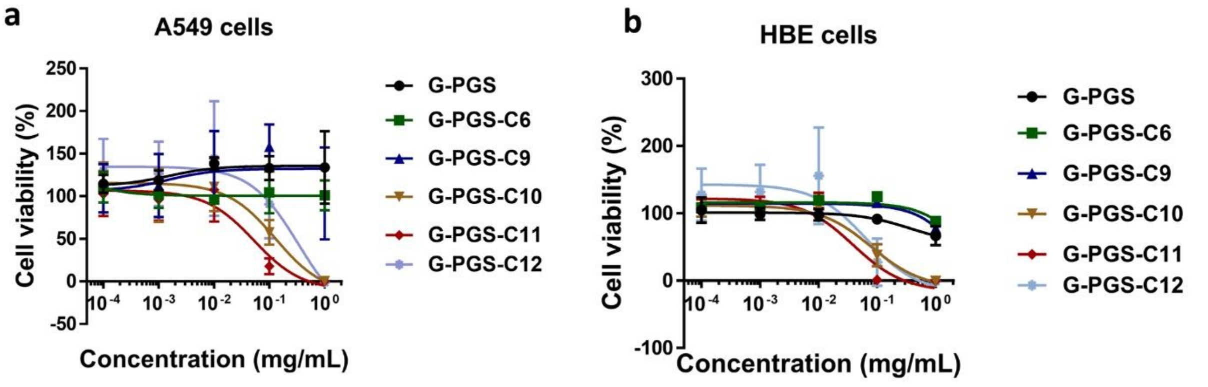

Figure S4. Cellular toxicity of the functional graphene platforms against HBE and A549 cells. Materials without or with short aliphatic chains (< 10) do not show toxicity to the cells, while their analogs with long aliphatic chains (≥ 10) are toxic to the cells.

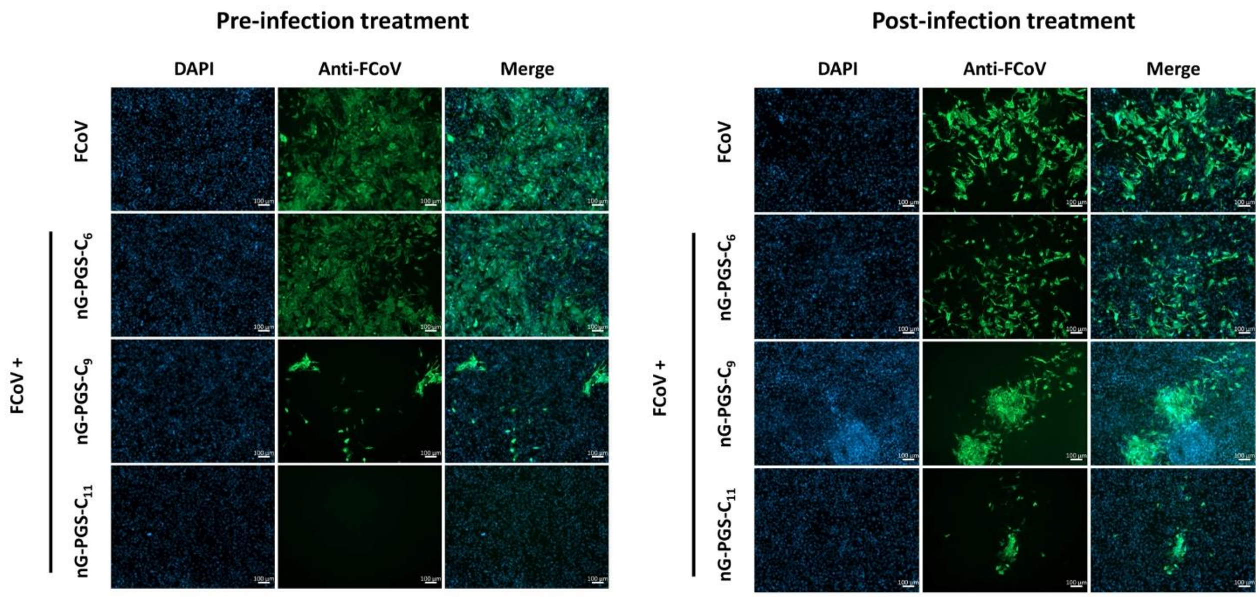

Figure S5. Immunofluorescent images for the FCoV infected cells in the presence of G-PGS-C6, G-PGS-C9, and G-PGS-C11 at 10 µg/mL. Scale bar: 100µm.

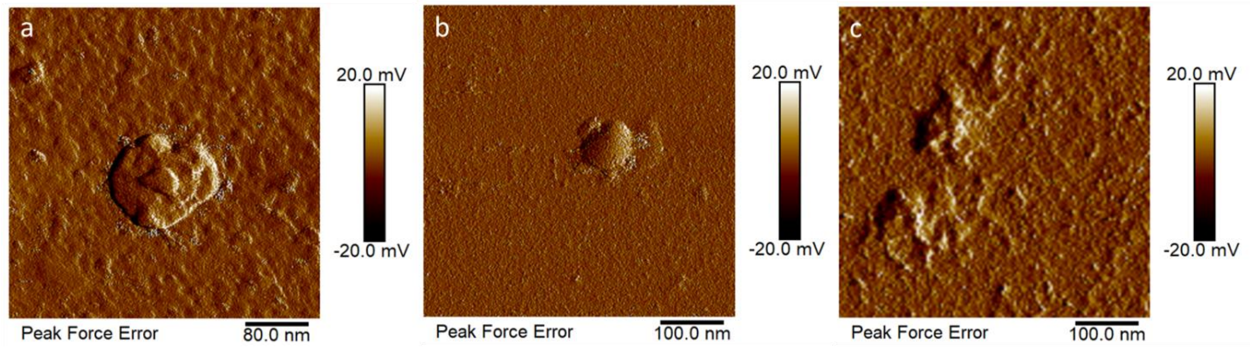

Figure S6. FCoV virions imaged by atomic force microscopy in Peak Force Error mode in PBS. (a) FCoV virion (b) FCoV virion mixed with G-PGS-C6. (c) FCoV virions mixed with G-PGS-C11 showing disrupted structures. Peak Force Error mode images clearly show the morphology of virions and their disruption in case of interactions with G-PGS-C11.

References

[1] A. Faghani, I. S. Donskyi, M. Fardin Gholami, B. Ziem, A. Lippitz, W. E. S. Unger, C. Böttcher, J. P. Rabe, R. Haag, M. Adeli, *Angewandte Chemie International Edition* **2017**, *56*, 2675-2679.
[2] H. Frey, R. Haag, *Reviews in Molecular Biotechnology* **2002**, *90*, 257-267.
[3] S. Roller, H. Zhou, R. Haag, *Molecular Diversity* **2005**, *9*, 305-316.
[4] I. S. Donskyi, W. Azab, J. L. Cuellar-Camacho, G. Guday, A. Lippitz, W. E. S. Unger, K. Osterrieder, M. Adeli, R. Haag, *Nanoscale* **2019**, *11*, 15804-15809.
